# Comparative Analysis of Waste, Steel, and Polypropylene Microfibers as an Additive for Cement Mortar

**DOI:** 10.3390/ma16041625

**Published:** 2023-02-15

**Authors:** Mateusz Zakrzewski, Mateusz Gancarz, Katarína Tvrdá, Joanna Laskowska-Bury, Jacek Domski

**Affiliations:** 1 Faculty of Civil Engineering, Environmental and Geodetic Sciences, Koszalin University of Technology, Sniadeckich 2, 75-453 Koszalin, Poland; 2 Faculty of Civil Engineering, Slovak University of Technology, Radlinského 2766/11, 810 05 Bratislava, Slovakia

**Keywords:** recycled steel fibers, SFRC, waste fibers, polypropylene fibers, crack propagation, 3PBT

## Abstract

This study presents the results of laboratory experiments conducted to determine the mechanical parameters for cement mortar with various quantities of waste fibers, polypropylene microfibers, and steel microfibers. Waste fibers were used as samples and obtained using an end-of-life car tire recycling process. For comparison, samples with the addition of steel and polypropylene microfibers were tested. The same degrees of fiber reinforcement were used for all types of fibers. Ultimately, 22 mixtures of cement mortar were prepared. The aim of this study is therefore to present and compare basic mechanical parameter values. Compressive strength, flexural strength, fracture toughness, and flexural toughness were of particular interest. A three-point bending test was performed on three types of samples, without a notch and with a notch of 4 and 8 mm. The results show that the use of steel microfibers in the cement mortar produces a product with better properties compared to a mixture with steel cord or polypropylene fibers. However, the cement mortar with the steel cord provides better flexural strength and greater flexural toughness factors compared to the cement mortar with polypropylene fibers. This means that the steel cord is a full-value ecological replacement for different fibers.

## 1. Introduction

At present, one of the main research topics studied by construction scientists is the use of recycled materials. One way to use waste materials in the production of concrete is to replace natural aggregate with materials that include, e.g., waste material obtained from the production of red ceramics and porcelain [1,2], cullet [3,4,5], mine waste [6,7,8], lathe [9,10], or others [11,12]. Another example of the use of materials derived from the waste recycling process is the use of waste as an additive to the concrete mix, improving its selected parameters, e.g., tightness [3,13], deadweight [14], and insulation properties [15]. Used car tires are a serious problem for the environment. Tires are non-biodegradable; therefore, recycling is very important. It is estimated that over one billion used tires exist annually, worldwide. Approximately one quarter of this number is created in the EU alone. Environmental regulations have a significant impact on the waste management of used tires. EU directives started banning the disposal of whole tires to landfills in July 2003, and also prohibited the disposal of tire by-products to landfills starting in 2006 [16,17]. In recent years, the number of tires used worldwide increased and is expected to increase further in the future. Tires constitute a waste product that requires particular treatment, as they can cause serious environmental and health issues [18]. The optimal solution seems to be the creation of a composite material composed of concrete and steel fibers from recycled tires.

To date, numerous researchers are studying the use of recycled fibers obtained from tires for concrete reinforcement [19,20,21] and presented numerous interesting conclusions. With the state of the economy of used tires at present, there remains a problem related to the rational management of waste, which is steel fiber. The search for an alternative product in the form of steel cord obtained from recycled tires and the study of its impact on the properties of the composite are necessary in the research [22,23,24]. The research conducted on steel cord fatigue was already performed and the results are promising. The type of bending (unidirectional is more favorable) and carbon content (a higher content is more favorable) has the greatest impact on the strength of the tested cord [25]. In their research, A. Simalti and A. P. Singh demonstrated the economic and environmental benefits of using recycled fibers. They obtained comparable flexural strength and split tensile strength in self-compacting concrete samples with recycled and serial-produced fibers [26]. K. Pilakoutas and others, in their article, presented precise information on the economics of the use of a cord. They compared the prices of reinforcing bars and commercially produced fibers. In their conclusions, the authors concluded that steel cord may be a cheaper replacement for commercial reinforcement [16]. Scientist M. Pajak noted in her research that the equivalent and residual flexural tensile strengths increased proportionally to the amount of fibers. The maximum flexural tensile strength and flexural strength were slightly improved by the fibers [27]. Interesting test results for the shear [28] and compression [29] qualities of fiber-reinforced concrete, derived from recycled tires, illustrated the wide range of applications of blends using recycled tire fibers. A geometric examination of the steel fibers confirmed that the fibers obtained from the recycling of tires used for the tests are characterized by varying lengths, diameters, slenderness, and shapes. Such a mixture can be classified as hybrid fibers, which show greater efficiency in concrete than fibers of equal lengths [30]. The concrete obtained by adding recycled steel fibers presents good mechanical improvement of the brittle matrix; as a consequence, it appears to be a promising candidate for both structural and non-structural applications [31]. According to the authors of articles [32,33,34], the addition of steel fibers positively influences the bending tensile strength of concrete samples. As demonstrated by the writers of the article [35], it is advantageous to use fibers with hooked ends—this improves the anchoring in the concrete matrix. Another important factor influencing the mechanical properties of reinforced concrete is the geometry of the fibers. Industrial steel fibers can be replaced by an equal (or slightly higher) number of recycled fibers without impairing the mechanical properties, provided that the recycled fibers have the appropriate geometrical characteristics [36]. When recycled concrete additives are successfully used, numerous benefits for the environment are recognized (the reduction in the carbon footprint, energy-saving aspect, and reduction in the amount of waste through their applications in a new material) [37,38,39].

In addition to steel fibers, recycled aggregates can also be used. Some recycled aggregates have a negative overall effect when the results of the concrete matrix test are observed; however, the addition of recycled fibers may eliminate the negative effect of recycled aggregates in the concrete. Studies showed that the thickness of the tested concrete slabs decreases with increasing the quantity of recycled fibers [40]. An additional possibility is the use of rubber as an additive to the aggregate. The addition of rubber deteriorates the mechanical properties of the matrix, but the addition of mass-produced steel fibers helps to compensate for these deficiencies [41]. It is assumed that it could be similar to the addition of steel fibers obtained from recycled tires and rubber as an aggregate additive; however, further research is necessary. Researchers A. Karimi and M. Nematzadeh investigated the behavior of concrete samples (surrounded by a steel pipe) with the addition of rubber obtained from recycled tires (with and without the addition of industrially produced fibers) under the influence of high temperatures. The dispersed reinforcement improved the compressive strength, despite the addition of rubber, which was degraded under the influence of the high temperature [42]. It can be assumed that the slight pollution of the steel fibers from the recycled tires with rubber will have a minor impact on the strength of the concrete elements. As the authors of the article [15] depict in this study, waste tires can also be used as building elements for walls and floors, for example. By using whole tires as an integral component, the carbon footprint of a new facility is reduced. The same result may be achieved with steel fiber-reinforced concrete obtained from recycled tires. The carbon footprint of objects using this form of concrete will be relatively smaller. The authors of the article, “Effect of Fiber Distribution on the Mechanical Behavior in Bending of Self-Compacting Mortars” [43], also achieved the improvement of the compressive and bending strengths of concrete mixes with the addition of industrially produced steel cord. Other tests conducted on 40 × 40 × 160 mm concrete samples with various amounts of polypropylene fibers proved the deterioration of the mechanical properties of the samples caused by a specific addition of fiber [44,45]. The best results were obtained for the addition of polypropylene fiber of approximately 1–1.5%, while with a higher amount, the strength values decreased [44]. In the case of the addition of recycled steel cord, as well as serially produced steel cord, the increase in the number of fibers in the mixture improved the compressive and bending strengths.

Polypropylene microfibers are another type of reinforcement that were used in the present study. They are organic fibers composed of artificial polymers. Polypropylene fibers are a good alternative to steel fibers, presenting a high tensile strength at a low weight. Polypropylene fibers weigh 900–910 kg [46]; for comparison, steel fibers weigh 7850 kg/m^3^. Polypropylene fibers are resistant to the chemical influence of the environment—the fibers are resistant to corrosion. Concrete reinforced with propylene fibers is used to produce items for use in public spaces (such items are exposed to adverse environmental conditions, impacts, surface abrasion, and vandalism), due to its anti-corrosion properties and increased strength [46]. The main disadvantage of polypropylene fibers is their low resistance to high temperatures. If the melting point of polypropylene fibers is exceeded, the fibers melt and create extra voids in the microstructure of the concrete. These extra voids negatively affect the mechanical strengths of the mortar, but can enhance spalling resistance [47]. The ingredients for the production of polypropylene fibers can be obtained from waste materials. The researchers [48] used recycled polypropylene fibers (produced from the cutting process of waste material) in their research, and achieved an increase in compressive, tensile, and compressive strength properties when splitting occurred (split tensile strength). The fiber content was tested in the range of 0.5–1.5%; the best results were obtained with 1.0%.

An understanding of the propagation of fatigue cracks in concrete is ever-increasing in the research. The literature presents the tests conducted on fatigue cracks in normal [49,50], high-strength [51], and self-compacting concretes [52], and concretes with the addition of ceramic waste [53]. In the present study, we investigate and compare the fatigue cracks in concrete with the addition of fibers obtained from recycled tires, steel fibers produced in series, and the addition of polypropylene fibers. In this paper, a three-point test of beams composed of a concrete mix with the addition of steel cords, steel, and polypropylene microfibers was used. Similar to the existing literature on the subject [2,53], beams with the dimensions of 40 mm × 40 mm × 160 mm were produced for the bending test, which were then used to determine the parameters of the cement mortar with the addition of reinforcement materials. Three types of beams were used: two beams with notches with depths of 4 and 8 mm, while seven mixtures with different reinforcement ratios were produced for each type of fiber. The following parameters were tested on these samples: flexural strength, flexural toughness, and fracture toughness. The standard EN 196-1 was used to prepare the cement mortar. In addition, the deflection in the center of the beam span was also measured, while a deflection speed of 2 mm/min was used. Following this test, the beam halves were used for compressive strength tests. Our present research is in line with the trend that is visible in the review publications on the possibility of using recycled tire fibers in construction [54,55,56,57].

In this article, waste fibers were analyzed. The steel cord used as a reinforcement in the following tests was obtained during the end-of-life car tire recycling process. The recovery of materials from used tires was performed either mechanically or through thermal degradation processes. The first method reduces the tires to steel fibers and granular rubber, and the second breaks the tires down into steel, carbonization, fluids, and gases. The processes of thermal degradation include cryogenic and pyrolysis processes. In the cryogenic process, the temperature of the tires is lowered and then the product is reduced to rubber and steel. The tires are cooled to below the brittle temperature. In the following step, the shredded tires are granulated and reduced to rubber, steel fibers, and textiles. In the process of pyrolysis, tires are thermally decomposed into organic and inorganic components (heated without oxygen). Pyrolysis is an energy efficient process, as the derived gases and oil have a high calorific value and can be used to meet the energy requirements of the process [16]. The amount of cord obtained mainly depends on the type of tire. Light-vehicle tires contain up to 15% steel, while truck tires contain up to 25% steel [17].

## 2. Materials and Methods

**Figure 1:**
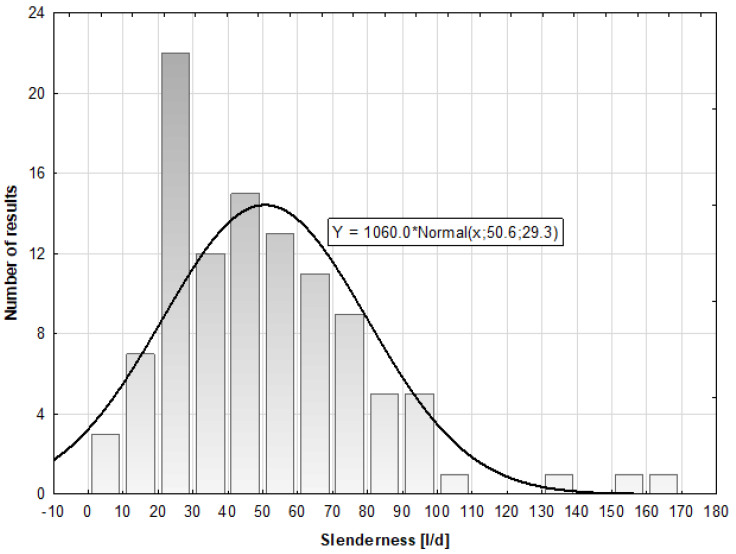
Slenderness of steel cord.

Before using the steel cord in the mixtures, its basic parameters were determined. Due to the different types and sizes of tires, the steel cord obtained varied in terms of the length and diameter of the fibers. In order to determine the general parameters of the cord, a sample consisting of 106 fibers randomly obtained from a bag supplied by the manufacturer was analyzed. The results are presented in Table 1. An important parameter describing the fibers is their slenderness, which is why, for the selected group of fibers, the numbers of fibers were classified into individual slenderness ranges (Figure 1).

The diameter of the majority of waste fibers (90%) is between 0.20 and 0.35 mm. The diameter of the fibers was measured with a micrometer. The tensile strength of fibers smaller than 0.30 mm in diameter could not be tested because they slid out of the jaws of the testing machine. Only samples ranging from 0.30 to 0.35 mm were used for tensile strength tests. The results are presented in Table 2. An additional advantage of the steel cord is its flexibility, which translates resistance into bending [58].

**Figure 2:**
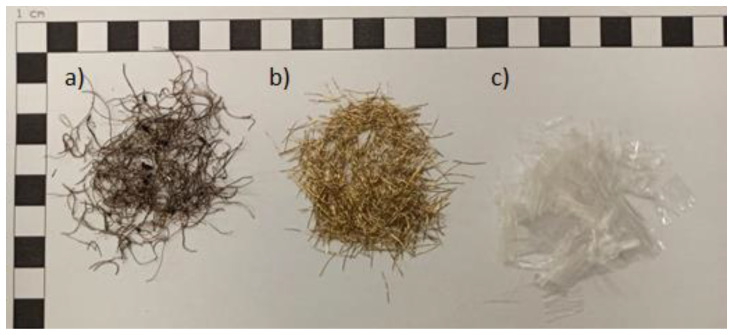
(**a**) Steel cord, (**b**) steel microfibers, and (**c**) polypropylene microfibers.

To compare the effect of the addition of the steel cord on the properties of cement mortar to commercial fibers, fibers of similar dimensions were selected (Figure 2). Steel microfibers with a length of 14 mm, diameter of 0.40 mm, and tensile strength of 1200 MPa were selected. Additionally, polypropylene microfibers with a length of 12 mm and tensile strength oscillating between the range of 600 and 650 MPa were also selected. The relative density of the fibers was 0.91 g/cm^3^. These data were provided by the manufacturers and were not verified by the authors of the present article.

**Figure 3:**
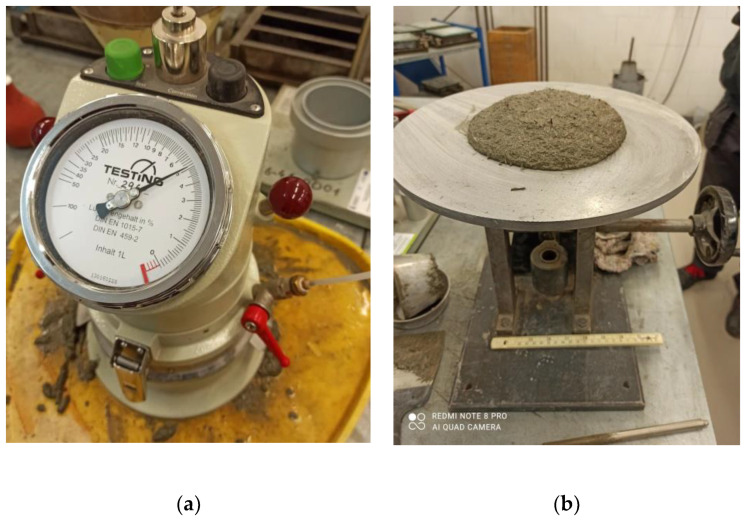
(**a**) Test of the air content in the fresh mixture, (**b**) test of the consistency of the fresh mixture: cone method.

The procedure described in standard EN 196-1 [59] was used to prepare the cement mortar. CEM I 42.5 R cement and standard sifted sand [59] were also used. For each type of fiber, specimens were created with fiber-reinforcement ratios of 0%; 0.25%; 0.5%; 0.75%, 1.0%; 1.25%; and 1.5%. For each type of ratio, three specimens were tested. A total of 189 specimens were tested for bending properties. The specimens were unmolded at 24 h and placed in a chamber with a constant temperature of 20 °C ± 1. The beams were tested at 28 days. During the preparation of the specimens, the air content in the fresh mixture was tested. The test was performed in accordance with EN 413:2005 (Figure 3a). The consistency of the fresh mixture was determined by the reed cone method based on the EN 1015-3:2000 standard (Figure 3b). The tests were performed for all mixtures. The results of the fresh mortar tests are presented in Table 3.

The following several parameters were used to determine the properties of the bent beams:

Flexural strength is calculated with the following equation:

(1) $$\sigma_{b}=\frac{3Pl}{2bh^{2}},$$

where:

$\sigma_{b}—$flexural strenght [N/mm^2^];

*P*—maximum load indicated by the testing machine until failure of the specimen [N];

*l*—span [mm];

*b*—width of faild coss-section [mm];

*h*—height of faild coss-section [mm].

**Figure 4:**
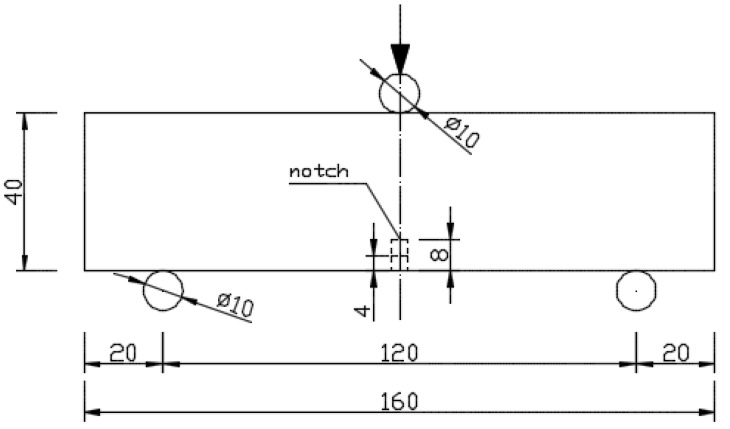
Setup for measurement of flexural strength.

The dimmensions were in accordance with Figure 4.

**Figure 5:**
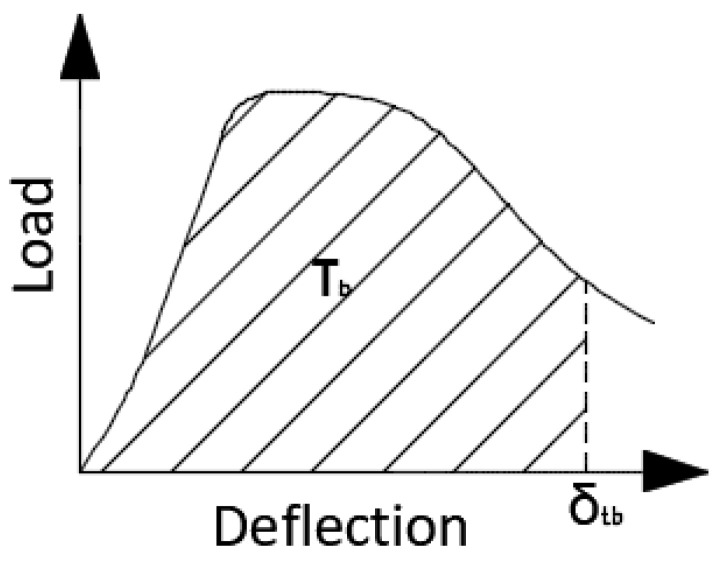
Flexural toughness.

Flexural toughness is determined by the area under the load-deflection curve until the measured deflection is 1/150 of the span (Figure 5) [60]. The flexural toughness factor is calculated using the following equation:

(2) $${\bar{\sigma }}_{b}=\frac{T_{b}}{\delta_{tb}}.\frac{l}{bh^{2}},$$

where:

${\bar{\sigma }}_{b}$—flexural toughness factor [N/mm^2^];

$T_{b}$—flexural toughness [J];

$\delta_{tb}$—deflection of 1/150 of span [mm].

An important aspect of concrete elements is the brittleness of the concrete matrix; the addition of fibers improves the brittleness of the concrete. In order to investigate the influence of tested fibers on the cracking properties of concrete, tests of the samples’ content were performed. Using the three-point bending method, the fracture toughness was examined. Fracture toughness is calculated using the three-point bending test data via the following equation:

$$K=\sigma \sqrt{\pi a}f\left(\frac{a}{W}\right)$$

where:

*a*—crack length;

*f(a/W)*—dimensionless geometry function for three-point bending test specimen [36,37,38].

$$f\left(\frac{a}{W}\right)=0.9926+0.4862\left(\frac{a}{W}\right)-12.479{\left(\frac{a}{W}\right)}^{2}+73.153{\left(\frac{a}{W}\right)}^{3}-124.29{\left(\frac{a}{W}\right)}^{4}$$

*σ*—value of load,

$$\sigma =\frac{3SP}{2tW^{2}}$$

where:

*S*—span [mm];

*P*—force [N];

*t*—thickness of specimen [mm];

*W*—width of specimen [mm].

## 3. Experimental Results

The following are the results that were obtained during the research and analysis conducted in the present study. The results of the fresh mortar tests are presented in Table 3.

**Figure 6:**
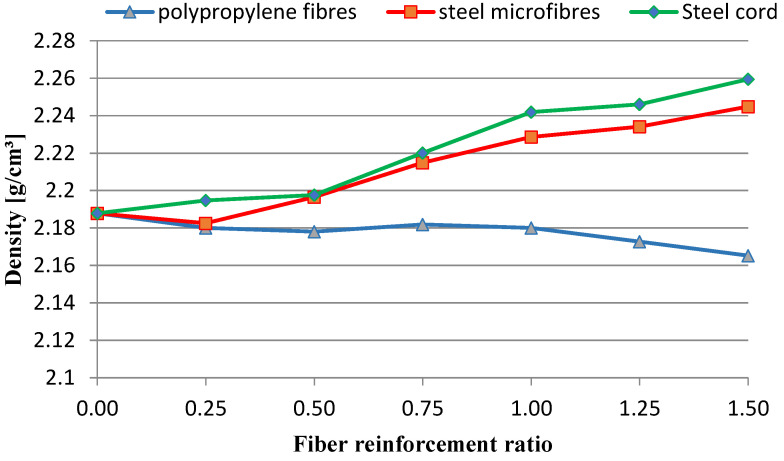
Density of tested mixtures.

The density of the tested mixtures was determined after 28 days. The specimens were measured and weighed. The results are presented in Figure 6.

Mechanical properties of the studied materials, tested in a traditional, constant manner, are reported in the following sections. The compressive strength, flexural strength, and flexural toughness were the subjects of interest in the study.

### 3.1. Flexural Strength

**Figure 7:**
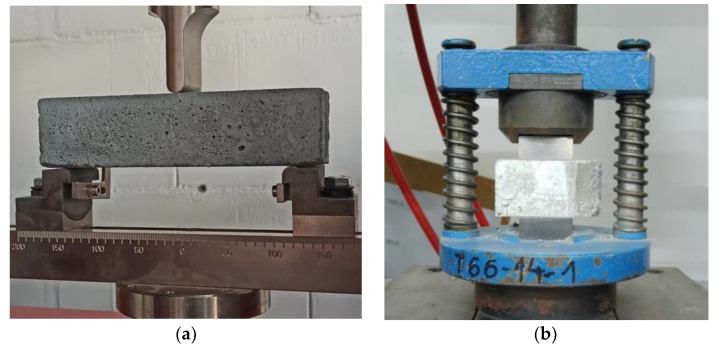
(**a**) Flexural strength and (**b**) compressive strength tests.

**Figure 8:**
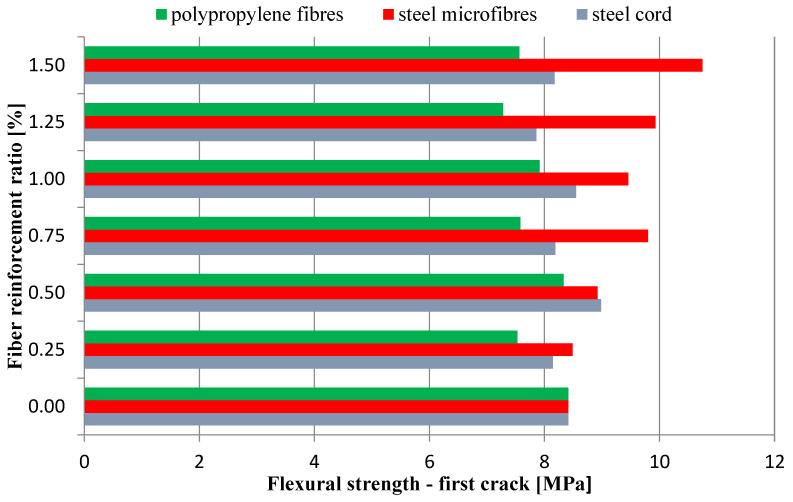
Flexural strength—first crack.

**Figure 9:**
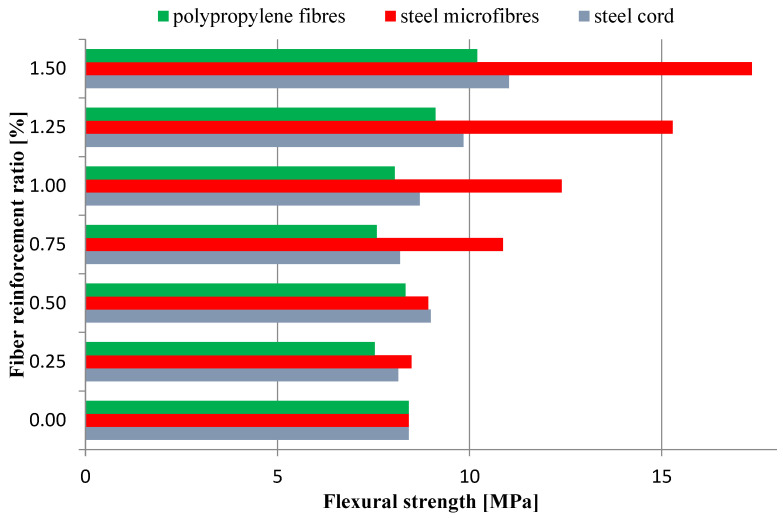
Flexural strength.

Three-point bending tests were performed on a universal testing machine with a sensor, with a range of 20 kN (Figure 7). The results obtained for the flexural strength tests are presented in Figure 8 and Figure 9, where the average values are shown. Figure 8 presents the flexural strength values determined for the force corresponding to the first crack.

Figure 9 presents the flexural strength values calculated in Equation (1).

**Figure 10:**
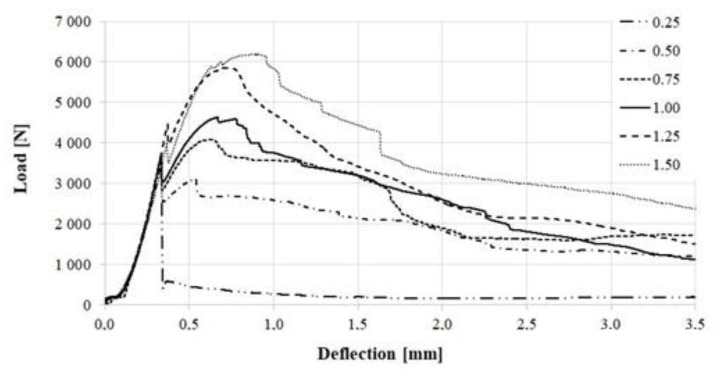
Load-deflection curve for specimens with steel cord.

**Figure 11:**
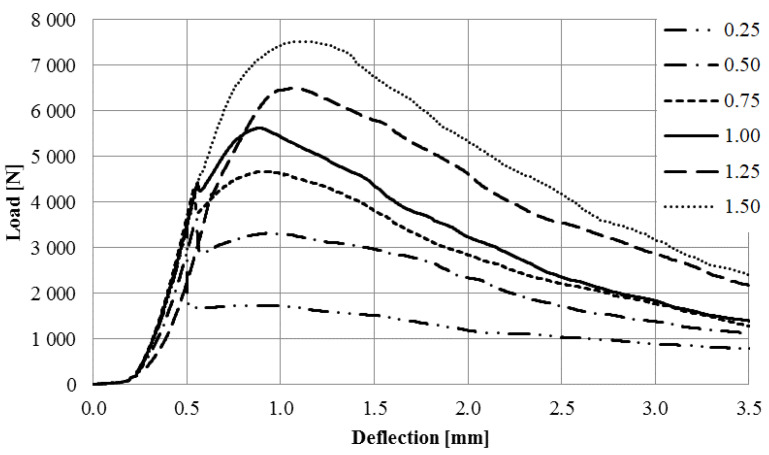
Load-deflection curve for specimens with steel microfibers.

**Figure 12:**
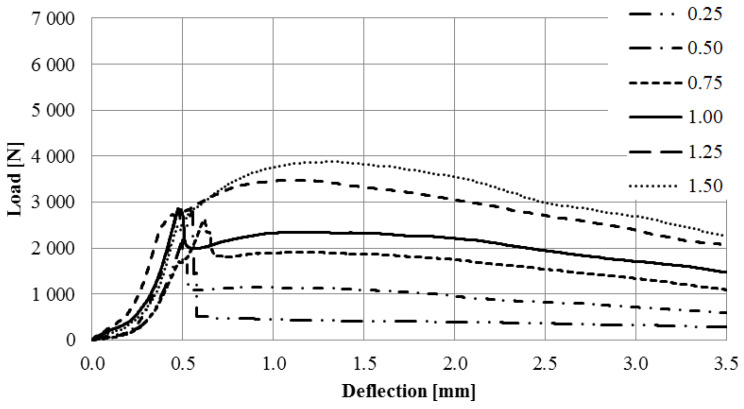
Load-deflection curve for specimens with polypropylene fibers.

Figure 10, Figure 11 and Figure 12 present the selected load-deflection curve for each fiber-reinforcement ratio. Figure 10 presents the curves for specimens with a steel cord.

Figure 11 presents the curve for specimens with steel microfibers.

Figure 12 presents curve for specimens with steel microfibers.

Based on the obtained load-displacement curves, the flexural toughness and flexural toughness factor were determined. After the bending test samples were divided into two parts, each part was used to perform the compressive strength test (Figure 7b). Table 4 shows the flexural toughness of specimens with steel and polypropylene fibers.

Table 5 presents the fracture toughness calculated for samples with notch values 4 and 8 mm.

### 3.2. Compressive Strength

**Figure 13:**
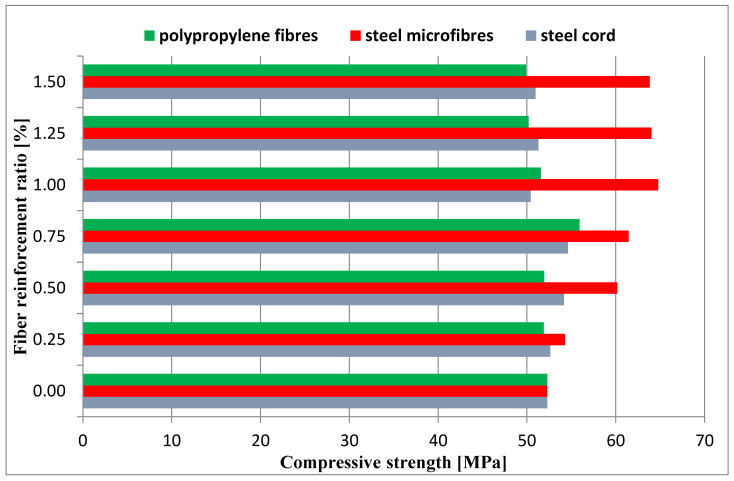
Compressive strength values of tested mixtures.

Compressive strength tests were also performed 28 days after the elements were created. The compressive strength test was conducted in compliance with [59] on halves of beams with the dimensions of 40 mm × 40 mm × 160 mm. The results obtained from the compressive strength tests are presented in Figure 13, where the average values are also presented.

## 4. Discussion

The aim of the study was to determine the effect of the addition of waste and commercial fibers on the selected mechanical parameters of cement mortar. When analyzing the results of the mixture consistency test, it should be stated that the steel cord had a lesser effect on the workability parameter compared to steel microfibers. At the highest content of fibers, the mixture with the steel cord, the measured flow diameter was 11.8% less compared to the material without fibers. For a mixture with the addition of steel microfibers, the difference was 17.4%. The density of both mixtures was almost identical. This is most likely due to the irregular shape of the fibers. The advantage of polypropylene fibers is that they slightly reduce the density of the material. The results obtained show that the addition of steel cord to concrete improves its compressive strength, depending on the fiber reinforcement ratio, from 12.85% to 22.18%. Studies show a significant decrease in the compressive strength of samples with the addition of polypropylene fibers. With an increase in the degree of dispersed reinforcement, the compressive strength decreases, except for samples with reinforcement at the level of 1.5%. However, the values for samples with fiber quantities of 1.0%, 1.25%, and 1.50% are very similar. The influence of steel cord on flexural strength values is dependent on the fiber reinforcement ratio. The higher the fiber reinforcement ratio, the greater the flexural strength. The maximum increase for a fiber reinforcement ratio of 1.5% is 68.37%. As for the case of compressive strength, the addition of polypropylene fibers caused a decrease in flexural strength. The decrease was between 15.34% and 22.00% of the value for non-fiber samples. An important factor is the fracture toughness of the specimen. It allows us to estimate the effectiveness of fiber reinforcement. The highest values of fracture toughness were recorded for specimens with steel microfibers. The higher the fiber reinforcement ratio, the higher the value. The maximum increase values compared to the fiber-free material were 149% (4 mm notch) and 85% (4 mm notch). For specimens with a steel cord, a significant increase of 88%/57% and 81%/32% (4 mm/8 mm notch) was noted for the fiber reinforcement ratios of 1.25 and 1.5, respectively. Mixtures with the addition of polypropylene fibers did not present significant changes in the fracture toughness value.

No plasticizer was used in the tests and the water–cement ratio was 0.5 for all mixtures. Due to the unusual shape of the cord, the use of a plasticizer may improve the consistency of the mixtures, and thus positively affect the parameters of mixtures with a higher fiber reinforcement ratio.

## 5. Conclusions

Tests performed on small samples with dimensions of 40 × 40 × 160 mm showed good properties of materials with steel cord reinforcements. The authors indicate the value of 0.75 as the optimal number of steel cord and steel microfibers. This number ensures a relatively high curve (Figure 5) whilst also considering economic considerations. Based on the research and analyses conducted, the following conclusions can be drawn:

- The steel cord is a full-value, ecological replacement for polypropylene fibers. Steel cord provides better flexural strength and a greater flexural toughness factor.
- The use of steel microfibers allows us to obtain better mixture properties than steel cord and polypropylene fibers.
- The use of steel cord as dispersed reinforcement will allow for improved rational waste management and a reduction in energy consumption values required for remelting the steel cord.
- The test conducted on small beams is a good method to determine the effect of fibers as an additive to concrete prior to starting tests in accordance with the standard EN 14651-2005.
- The use of a plasticizer may improve the consistency of the mixtures with steel cord, and thus positively affect the parameters of mixtures with a higher fiber reinforcement ratio.

Materials with cord reinforcements can be used, for example, to produce foundation slabs. This will allow for less usage of traditional reinforcement bars, which will decrease the time of work for preparing the reinforcement. In addition, it will reduce the cost of materials.

The future research on the properties of concrete with steel cord as dispersed reinforcement should focus on rheological properties. They are of significant importance for the application of this material in practice. Further research will indicate the optimal amount of fiber addition that is necessary.

## Figures and Tables

**Table 1:** Parameters of steel cord.

Parameter	Average	Standard Deviation	Median	Minimum Value	Maximum Value
	[mm]	[mm]	[mm]	[mm]	[mm]
diameter	0.31	0.18	0.25	0.05	1.35
length	13.29	5.80	12.57	2.90	27.90

**Table 2:** Tensile strength of steel cord.

Diameter	Average Tensile Strength	Standard Deviation	Coefficient of Variation
[mm]	[MPa]	[MPa]	[%]
0.30–0.35	1788.53	543.88	0.30

**Table 3:** Fresh mixture test results.

Fiber Reinforcement Ratio	Steel Cord		Steel Microfibers		Polypropylene Fibers	
[%]	Air Content	Consistency	Air Content	Consistency	Air Content	Consistency
	[%]	[cm]	[%]	[cm]	[%]	[cm]
0.00	6.2	17,8	6.2	17,8	6.2	17,8
0.25	6.0	17.5	5.9	17.3	6.2	17.0
0.50	5.5	16.8	6.0	17.0	6.0	15.5
0.75	5.2	16.5	5.7	16.3	5.7	15.2
1.00	5.3	16.2	5.4	15.7	5.8	15.2
1.25	5.0	16.0	5.0	14.7	5.0	14.5
1.50	4.9	15.7	4.2	14.7	4.8	13.0

**Table 4:** Flexural toughness factor [60].

Fiber Reinforcement Ratio	Steel Cord	Standard Deviation	Steel Microfibers	Standard Deviation	Polypropylene Fibers	Standard Deviation
[%]	[MPa]	[MPa]	[MPa]	[MPa]	[MPa]	[MPa]
0.25	1.41	0.40	2.44	0.23	1.18	0.05
0.50	3.14	0.33	3.24	0.15	1.41	0.11
0.75	4.11	0.27	3.98	0.19	1.44	0.35
1.00	3.86	0.31	4.22	0.12	1.92	0.14
1.25	4.91	0.69	4.21	0.48	2.64	0.32
1.50	4.74	0.36	4.62	0.28	2.09	0.01

**Table 5:** Fracture toughness.

Fiber Reinforcement Ratio	Fracture Toughness Steel Cord		Fracture Toughness Steel Microfibers		Fracture Toughness Polypropylene Fibers	
notch	4 mm	8 mm	4 mm	8 mm	4 mm	8 mm
[%]	[MPa]	[MPa]	[MPa]	[MPa]	[MPa]	[MPa]
0.00	0.809	1.085	0.664	1.082	0.647	1.056
0.25	0.804	1.173	0.708	1.123	0.573	1.090
0.50	0.768	1.121	0.819	1.313	0.625	1.050
0.75	0.885	1.392	1.084	1.437	0.694	1.077
1.00	0.991	1.356	1.366	2.040	0.651	1.124
1.25	1.523	1.704	1.616	2.006	0.679	1.174
1.50	1.470	1.438	1.655	1.846	0.706	1.111

## Data Availability

The data presented in this study are available on request from the corresponding author.
